# Magnesium Status in Celiac Disease: Potential Links with Neuroinflammatory Pathways and Gut–Brain Axis Dysfunction

**DOI:** 10.3390/ijms27146200

**Published:** 2026-07-11

**Authors:** Elena Popa, Andrei Emilian Popa, Mihaela Poroch, Vladimir Poroch, Alexandru Matei Hatneanu, Ana-Maria Slanina, Monica Iuliana Ungureanu, Tatiana Dramba, Antoneta Dacia Petroaie, Agnes Iacinta Bacusca, Gema Bacoanu, Elena Adorata Coman

**Affiliations:** 1 Faculty of Medicine, “Grigore T. Popa” University of Medicine and Pharmacy, 16 Universitatii Str., 700115 Iasi, Romania; elena.popa@umfiasi.ro (E.P.); boanca.mihaela@umfiasi.ro (M.P.); vladimir.poroch@umfiasi.ro (V.P.); hatneanualex@gmail.com (A.M.H.); ana_slanina@umfiasi.ro (A.-M.S.); monica.ungureanu@umfiasi.ro (M.I.U.); tatiana.dramba@email.umfiasi.ro (T.D.); antoneta.petroaie@umfiasi.ro (A.D.P.); bacusca.agnes@umfiasi.ro (A.I.B.); gema.bacoanu@umfiasi.ro (G.B.); elena.coman@umfiasi.ro (E.A.C.); 2 Department of Family Medicine, Preventive Medicine and Interdisciplinary, “Grigore T. Popa” University of Medicine and Pharmacy, Universitatii Str. 16, 700115 Iasi, Romania; 3 “Prof. Dr. Nicolae Oblu” Emergency Clinic Hospital, 700309 Iasi, Romania; 4 2nd Internal Medicine Department, “Grigore T. Popa” University of Medicine and Pharmacy, 700115 Iasi, Romania

**Keywords:** celiac disease, magnesium, neuroinflammation, gut–brain axis, gluten-free diet, neurological manifestations

## Abstract

Celiac disease (CD) is a chronic immune-mediated enteropathy characterized by intestinal inflammation, epithelial barrier dysfunction, and a wide range of extraintestinal manifestations, including neurological disorders. Magnesium (Mg) status has attracted increasing interest because Mg is involved in inflammatory regulation, oxidative balance, mitochondrial function, calcium homeostasis, and neuronal signaling, suggesting a potential role in neuroimmune pathways and gut–brain axis communication. This narrative review critically evaluates the available evidence regarding the relationship between Mg status, neuroinflammation, and gut–brain axis dysfunction in CD. A literature search was performed using PubMed, Scopus, and Web of Science, focusing primarily on studies published between 2019 and 2025, while earlier landmark publications were included to provide mechanistic context. The review was conducted according to the Scale for the Assessment of Narrative Review Articles (SANRA). Current evidence indicates that reduced Mg status in CD is primarily associated with intestinal malabsorption, chronic inflammation, and dietary inadequacies. Experimental and translational studies support several mechanisms through which Mg may influence neuroimmune signaling; however, clinical evidence directly linking altered Mg status to neurological manifestations in patients with CD remains limited. Overall, Mg should be regarded as a potential modifier of inflammatory and neuroimmune pathways rather than an independent pathogenic factor. Further prospective studies are needed to clarify the contribution of Mg homeostasis to neurological manifestations in CD.

## 1. Introduction

Celiac disease (CD) is a chronic immune-mediated enteropathy triggered by gluten exposure in genetically predisposed individuals and affecting approximately 1% of the global population [1]. It is characterized by intestinal inflammation, villous atrophy, and impaired epithelial barrier function [2,3]. Although traditionally regarded as a gastrointestinal disorder, CD is now recognized as a systemic condition with a broad spectrum of extraintestinal manifestations involving the hematological, musculoskeletal, dermatological, endocrine, hepatic, and nervous systems [4,5,6,7]. Neurological and neuropsychiatric manifestations—including peripheral neuropathy, gluten ataxia, headache, cognitive complaints, anxiety, and depressive symptoms—have attracted increasing attention, suggesting that intestinal immune activation may exert effects beyond the gastrointestinal tract [5,6,7,8,9].

The mechanisms underlying neurological involvement in CD are likely multifactorial and incompletely understood. Current evidence suggests that chronic systemic inflammation, circulating cytokines, increased intestinal permeability, dysbiosis, immune-mediated neuronal injury, and micronutrient deficiencies secondary to malabsorption or long-term dietary restriction may collectively contribute to nervous system involvement [8,9]. These processes can be integrated within the concept of the gut–brain axis, a bidirectional network linking the gastrointestinal tract and the central nervous system (CNS) through immune, neural, endocrine, and microbial pathways [9]. Persistent mucosal inflammation and epithelial barrier dysfunction may facilitate the systemic dissemination of inflammatory mediators and microbial products, thereby promoting neuroimmune communication and neuroinflammatory signaling [9]. However, the relative contribution of these interconnected mechanisms to neurological manifestations in patients with CD has yet to be established [9].

Among the nutritional abnormalities associated with CD, magnesium (Mg) deserves particular attention. Mg is an essential intracellular cation involved in ATP-dependent metabolism, membrane stability, calcium homeostasis, neurotransmitter release, synaptic function, and neurovascular regulation [10,11,12,13,14]. It also modulates inflammatory signaling, oxidative balance, mitochondrial function, and neuronal excitability, all of which are relevant to pathways implicated in neuroinflammation and gut–brain axis communication [10,11,12,13,14]. In patients with CD, Mg status may be influenced from several converging factors, including intestinal malabsorption, chronic diarrhea, persistent low-grade inflammation, and inadequate dietary intake associated with long-term adherence to a gluten-free diet (GFD) [10,11,12,13,14]. Although not universally observed, reduced circulating Mg concentrations and suboptimal dietary intake have been reported in some individuals with CD [10,11,12,13,14].

Despite growing research interest, the role of Mg in the neurological manifestations of CD remains incompletely characterized. Much of the available literature describing Mg-dependent neuroimmune mechanisms derives from experimental models or studies conducted in non-celiac inflammatory and neurodegenerative disorders [9,15,16,17]. Although these investigations provide valuable mechanistic insight, their clinical relevance in patients with CD has yet to be established. Consequently, whether variations in Mg status contributes directly to neuroinflammatory pathways or neurological manifestations in CD has yet to be determined [6,9,15,16,17].

Accordingly, this structured narrative review critically synthesizes the available evidence regarding Mg status in CD and its relationship with neuroinflammatory pathways and gut–brain axis communication. Particular emphasis is placed on altered Mg homeostasis, systemic inflammation, and the mechanisms through which Mg may modulate neuroimmune signaling, while critically distinguishing experimental and translational evidence from the currently available clinical data.

## 2. Literature Search

This structured narrative review was based on a comprehensive literature search conducted in PubMed, Scopus, and Web of Science using combinations of the following keywords: “celiac disease”, “magnesium”, “neuroinflammation”, “gut–brain axis”, “gluten-free diet”, and “neurological manifestations”.

The primary search covered publications from 2019 to 2025, while earlier landmark articles were included when necessary to provide physiological and pathophysiological context.

Eligible publications included experimental, translational, observational, and clinical studies, together with narrative and systematic reviews investigating magnesium homeostasis, intestinal inflammation, neuroimmune mechanisms, oxidative stress, mitochondrial dysfunction, neurotransmitter regulation, and gut–brain axis dysfunction in celiac disease. Priority was given to peer-reviewed human studies, systematic reviews, meta-analyses, and mechanistic studies relevant to the objectives of this review.

To enhance methodological transparency and reporting quality, this review was prepared in accordance with the Scale for the Assessment of Narrative Review Articles (SANRA) [18]. The SANRA criteria were considered throughout manuscript preparation to ensure transparent literature selection, balanced presentation of the available evidence, appropriate referencing, scientific reasoning, and critical interpretation of the published data.

## 3. Celiac Disease as a Systemic Inflammatory and Immune-Mediated Disorder

CD is a systemic immune-mediated disorder triggered by dietary cereal prolamins in genetically predisposed individuals and characterized by gluten-dependent symptoms, disease-specific autoantibodies, HLA-DQ2/DQ8 haplotypes, and enteropathy [19]. Although traditionally defined by its gastrointestinal manifestations, CD frequently presents extraintestinal complications resulting from chronic inflammation, immune dysregulation, and malabsorption, affecting multiple organ systems [19].

Untreated CD is further associated with autoimmune comorbidities, including type 1 diabetes mellitus, dermatitis herpetiformis, and autoimmune thyroid disease, as well as, in selected cases, long-term complications such as small intestinal adenocarcinoma and enteropathy-associated T-cell lymphoma [20]. Neurological and neuropsychiatric manifestations have also been reported, although their pathophysiological mechanisms remain incompletely understood [16,21].

### 3.1. Intestinal Inflammation and Immune Activation

Gluten proteins, particularly gliadin and glutenin fractions, contain proline- and glutamine-rich sequences that resist complete gastrointestinal proteolysis and generate immunogenic peptides, including the 33-mer α-gliadin peptide [8,19,22]. In genetically predisposed individuals, these peptides cross the intestinal epithelium and are deamidated by tissue transglutaminase 2 (TG2), enhancing their affinity for HLA-DQ2/DQ8 molecules and promoting activation of gluten-specific CD4^+^ T lymphocytes [19,21,23]. The resulting adaptive immune response is predominantly Th1-mediated and characterized by increased production of pro-inflammatory cytokines, including interferon-γ (IFN-γ), tumor necrosis factor-α (TNF-α), interleukin-17 (IL-17), and IL-15, leading to intraepithelial lymphocyte activation, enterocyte damage, villous atrophy, and epithelial barrier dysfunction [20,21,23,24].

In parallel, innate immune activation, driven by gliadin exposure, epithelial stress, and cytokine release, further amplifies mucosal inflammation [21,22,24]. Although anti-TG2 antibodies represent the principal serological hallmark of CD, B cells may also participate in disease pathogenesis through antigen presentation and by amplifying adaptive immune responses [22,24]. Overall, these immune mechanisms sustain chronic intestinal inflammation, drive systemic immune activation, and provide a mechanistic basis for the extraintestinal manifestations of CD.

### 3.2. Increased Intestinal Permeability and Microbial Translocation

Impaired epithelial barrier integrity is a hallmark of CD and facilitates the passage of gliadin-derived peptides and luminal antigens into the lamina propria, promoting mucosal immune activation [25]. Tight junction dysfunction altered expression of junctional proteins, and gliadin-induced zonulin release contribute to increased intestinal permeability, thereby enhancing immune exposure to dietary and microbial antigens [24,25,26,27,28]. Experimental evidence suggests that zonulin-mediated signaling promotes tight junction disassembly, whereas the resulting inflammatory milieu promotes villous atrophy, impaired nutrient absorption, and progressive epithelial barrier disruption [27,28,29]. Persistent cytokine-mediated inflammation, including increased expression of IL-15, TNF-α, IL-6, and IL-17, further amplifies epithelial injury and intraepithelial lymphocyte activation [28].

In addition to gluten exposure, dietary and microbial factors may influence intestinal barrier integrity and immune responses. Alterations in gut microbiota composition, infections, and microbial transglutaminase have been implicated in mucosal immune activation and epithelial barrier dysfunction, particularly in genetically predisposed individuals [21,23,25,26,27,30]. These interconnected processes sustain chronic intestinal inflammation, perpetuate barrier dysfunction, and facilitate systemic immune activation, thereby contributing to the extraintestinal manifestations of CD.

### 3.3. Systemic Inflammation and Circulating Cytokine Profile

Although CD originates in the intestinal mucosa, accumulating evidence indicates that its pathophysiology extends beyond the gastrointestinal tract and involves both local and systemic immune activation [30,31]. Gluten-driven immune responses activate innate and adaptive immune pathways, leading to the production of pro-inflammatory cytokines that propagate inflammatory signaling beyond the intestinal mucosa [31]. High-dimensional immune profiling has demonstrated remodeling of the lamina propria, altered macrophage populations, and transcriptional changes affecting both CD4^+^ and CD8^+^ T-cell compartments, highlighting the complexity of immune dysregulation in CD [23,31].

These local immune events are reflected in the systemic circulation. Gluten challenge studies in patients with CD adhering to a GFD have demonstrated rapid increases in circulating cytokines, including IL-2, IL-17A, IL-22, and IFN-γ, which correlate with symptom onset and further support the systemic nature of the gluten-induced immune response [32]. Sustained cytokine signaling provides a mechanistic basis for the development of extraintestinal manifestations, reinforcing the concept of CD as a multisystem immune-mediated disorder rather than a condition confined to the intestinal mucosa [23,31,32,33].

Beyond neurological involvement, systemic immune dysregulation contributes to a broad spectrum of extraintestinal manifestations. Hematological abnormalities, particularly iron deficiency anemia and folate or vitamin B12 deficiency, are among the most common manifestations. Musculoskeletal complications include osteopenia, osteoporosis, reduced bone mineral density, arthralgia, and impaired muscle function secondary to intestinal malabsorption and chronic inflammation. CD is also associated with autoimmune thyroid disease, type 1 diabetes mellitus, autoimmune liver disorders, and dermatitis herpetiformis, reflecting the multisystem consequences of sustained immune dysregulation [19,20,23,31,32,33].

These systemic immune alterations provide a mechanistic framework linking intestinal inflammation with neuroimmune communication and gut–brain axis signaling, thereby establishing the basis for the neuroinflammatory mechanisms discussed in the following sections.

### 3.4. Neurological and Neuropsychiatric Manifestations of CD

Neurological and neuropsychiatric manifestations represent some of the most clinically relevant extraintestinal complications of CD and may affect both the central and peripheral nervous systems [6,15]. These manifestations have been reported in both untreated and treated patients [32]. The clinical spectrum includes gluten ataxia, peripheral neuropathy, epilepsy, headache, migraine, cognitive impairment, and neuropsychiatric symptoms, although their prevalence and severity vary considerably across studies [6,9,15,32].

The pathophysiological mechanisms underlying neurological dysfunction are complex and likely multifactorial. Chronic systemic inflammation, cytokine-mediated signaling, intestinal barrier dysfunction, and immune dysregulation are considered major contributors to neuroinflammation and impaired gut–brain axis communication [9,33,34]. Increased intestinal permeability may facilitate BBB dysfunction and the systemic dissemination of inflammatory mediators, whereas circulating autoantibodies directed against tissue transglutaminase and neuronal antigens, particularly transglutaminase 6 (TG6), have been implicated in immune-mediated neuronal injury and gluten ataxia [9,17,35].

Among the neurological complications associated with CD, gluten ataxia is one of the best-characterized immune-mediated disorders related to gluten sensitivity [36,37]. Peripheral neuropathy is also frequently reported, and epidemiological studies suggest that individuals with CD have an increased risk of neuropathic disorders, including chronic inflammatory demyelinating neuropathy and autonomic neuropathy [38]. Epilepsy, headache, and migraine have likewise been reported more frequently in patients with CD than in the general population, particularly in pediatric cohorts and selected epilepsy phenotypes [15,39,40,41,42].

Cognitive and neuropsychiatric manifestations, including memory impairment, reduced attention, executive dysfunction, mental fatigue, “brain fog”, anxiety, and depressive symptoms, have also been described in CD, although their prevalence and underlying mechanisms are not yet fully understood [15,43,44,45]. Factors that may contribute include chronic inflammatory signaling, immune dysregulation, neurotransmitter imbalance, micronutrient deficiencies secondary to malabsorption, and psychosocial factors associated with long-term adherence to a GFD [15,44,45]. Possible associations with neurodegenerative disorders, including Alzheimer’s disease and vascular dementia, have also been reported; however, available evidence remains heterogeneous and does not support a causal relationship [46,47].

Experimental and translational studies suggest that inflammatory signaling may influence brain regions involved in affective regulation, including the anterior cingulate cortex, potentially contributing to anxiety and depressive symptoms [44,45,48]. However, these observations derive predominantly from broader neuroinflammatory research, and their relevance to CD has yet to be established.

Micronutrient deficiencies secondary to intestinal malabsorption may further influence neurological and neuropsychiatric manifestations in CD. Deficiencies of iron, folate, vitamin B12, vitamin D, vitamin E, and other micronutrients have all been implicated in neurological dysfunction, although their relative contribution varies according to the clinical presentation and disease severity [9,44,45,49]. These findings support the concept that neurological dysfunction in CD is multifactorial and reflects the combined effects of immune dysregulation, chronic inflammation, impaired intestinal barrier function, and nutritional disturbances rather than a single pathogenic mechanism.

## 4. Mg Homeostasis in Celiac Disease

### 4.1. Physiological Roles of Mg in Metabolism and Nervous System Function

Mg is the second most abundant intracellular cation and an essential cofactor for numerous enzymatic reactions involved in energy metabolism, ATP production, ion transport, nucleic acid synthesis, and intracellular signaling [11,50,51]. Through its role in ATP-dependent enzymatic reactions, Mg is required for Na^+^–K^+^-ATPase activity, maintenance of transmembrane ion gradients, and preservation of cellular membrane stability [50]. Consequently, intracellular Mg deficiency may result in intracellular accumulation of Na^+^ and Ca^2+^, K^+^ loss, and impaired cellular homeostasis [50]. Because of its fundamental role in cellular metabolism, immune regulation, and nervous system function, Mg has been increasingly investigated as a potential modulator of inflammatory and neuroimmune pathways in chronic inflammatory disorders, including CD [11,51].

Within the nervous system, Mg contributes to neuronal ion homeostasis, synaptic plasticity, and neurotransmitter release [10]. One of its best-characterized neuroprotective actions is the physiological regulation of N-methyl-D-aspartate (NMDA) receptors, through which Mg limits excessive glutamate-mediated Ca^2+^ influx, thereby reducing excitotoxicity and neuronal injury [52]. Reduced Mg availability has also been associated with increased substance P concentrations, oxidative stress, chronic inflammation, and enhanced neuronal excitability [10,53,54,55,56,57]. At the molecular level, Mg modulates inflammatory signaling partly through inhibition of nuclear factor kappa B (NF-κB), thereby reducing the expression of pro-inflammatory cytokines such as TNF-α and IL-6 [13,53]. Supporting these observations, a meta-analysis of randomized controlled trials suggested that Mg supplementation may improve inflammatory status, mainly through reductions in C-reactive protein (CRP) and plasma fibrinogen, whereas effects on individual cytokines remain less consistent [56].

Although Mg is not considered a classical antioxidant, it indirectly supports antioxidant defense by stabilizing superoxide dismutase (SOD) and maintaining mitochondrial ATP synthesis and calcium homeostasis, mechanisms relevant to oxidative balance, neuronal viability, and neuroinflammatory regulation [43,58,59]. Preclinical evidence further supports its neuroprotective properties through intracellular actions within mitochondria, nuclei, and the endoplasmic reticulum, where it promotes neuronal plasticity, energy metabolism, and resistance to oxidative stress [49,51,60]. Additional experimental studies have reported beneficial effects on cognitive performance, synaptic plasticity, oligodendrocyte function, and myelin integrity under conditions of cellular stress [10,60,61,62].

Reduced Mg availability has been associated with anxiety, depression, headache, and cognitive dysfunction, although much of the available evidence is derived from experimental models or indirect observations [51,60,62,63]. In patients with CD, chronic diarrhea and intestinal malabsorption may contribute to reduced Mg status [64]. Consequently, disturbances in Mg homeostasis have been proposed as potential modifiers of systemic inflammation and neuroimmune processes in chronic inflammatory and malabsorptive disorders [11,53]. Similar biological pathways have also been described in other inflammatory conditions, including long COVID, in which reduced Mg status has been associated with persistent inflammation, oxidative stress, mitochondrial dysfunction, endothelial dysfunction, and neurological symptoms such as fatigue, cognitive impairment, and headache [53,65,66]. Although these observations support the mechanistic rationale for a modulatory role of Mg in neuroimmune regulation, whether comparable pathways operate in patients with CD remains to be clarified.

Accordingly, extrapolation of findings from other inflammatory disorders should be interpreted cautiously until confirmed by well-designed clinical studies in patients with CD.

### 4.2. Determinants of Mg Status in Celiac Disease

The total body Mg pool is approximately 22–23 g, with nearly 60% stored in bone [65,66]. Under physiological conditions, dietary Mg is absorbed primarily in the small intestine, whereas renal reabsorption maintains plasma concentrations within a narrow physiological range. Because less than 1% of total body Mg is present in the extracellular compartment, serum Mg concentrations may not accurately reflect whole-body Mg stores and should therefore be interpreted with caution when evaluating Mg status [67,68]. This limitation should also be considered when interpreting studies assessing Mg homeostasis in CD.

Micronutrient deficiencies are frequently observed in patients with CD, particularly at diagnosis and in untreated disease. Reduced circulating concentrations of iron, folate, vitamin B12, vitamin D, zinc, and Mg have all been documented, reflecting the combined effects of intestinal malabsorption, mucosal damage, and chronic inflammation [64,69]. Lower serum Mg concentrations have been identified in a subset of individuals with CD [64,70]. At diagnosis, Mg deficiency has been reported in approximately 13–17% of untreated adults and 7–11% of untreated children [64]. Following long-term adherence to a GFD, persistent Mg deficiency has been observed in approximately 4% of pediatric patients, whereas data regarding long-term Mg homeostasis in treated adults remain scarce [70].

However, available clinical studies have yielded heterogeneous findings. A recent cross-sectional study evaluating multiple micronutrients in newly diagnosed patients with CD found no statistically significant differences in serum Mg concentrations compared with healthy controls [71]. These findings indicate that Mg deficiency is not a universal feature of CD and that its prevalence may vary according to disease stage, degree of mucosal recovery, dietary adherence, population characteristics, and the methods used to evaluate Mg homeostasis [64,70,71].

Suboptimal dietary Mg intake may further contribute to disturbances in Mg homeostasis in individuals adhering to GFD. Previous studies suggest that a substantial proportion of both adults and children consume less Mg than recommended, although these estimates are heterogeneous and derive predominantly from indirect or review-based evidence [64]. Population-based analyses likewise indicate lower Mg intake and an overall less favorable micronutrient profile among individuals with CD or gluten-related disorders [72,73].

These observations suggest that disturbances in Mg homeostasis are likely to reflect the combined effects of intestinal malabsorption, persistent low-grade inflammation, and inadequate dietary Mg intake associated with long-term GFD use. Experimental, translational, and observational studies have linked these alterations to inflammatory and neuroimmune pathways [11,53,56,73,74].

Nevertheless, considerable heterogeneity persists regarding both the prevalence and the clinical significance of Mg deficiency in CD [64,70,71]. Interpretation of Mg status should therefore be individualized and integrated into the overall clinical, nutritional, and disease context of each patient. Further prospective disease-specific studies are warranted to investigate the potential contribution of disturbances in Mg homeostasis to neurological outcomes, neuroinflammatory pathways, and gut–brain axis dysfunction in CD.

## 5. Interaction Between Neuroinflammation and Mg in Celiac Disease

Current knowledge indicates that Mg may influence several biological processes implicated in neuroinflammation and gut–brain axis dysfunction, including inflammatory signaling, oxidative stress, mitochondrial function, endothelial integrity, and neuronal excitability [11,53]. In CD, these pathways are of particular interest because persistent systemic inflammation, epithelial barrier dysfunction, and micronutrient deficiencies may collectively contribute to neurological manifestations [11,64]. However, direct clinical information linking Mg homeostasis with these mechanisms in patients with CD remains comparatively limited.

To facilitate critical appraisal of the available data, published reports were categorized according to study design, disease specificity, and translational applicability (Table 1). This framework differentiates observations obtained directly in CD populations from mechanistic findings derived from preclinical models or other inflammatory disorders, thereby providing a clearer perspective on their potential relevance to celiac disease.

Table 1 illustrates that the available studies primarily indicate the biological plausibility of Mg-dependent neuroimmune mechanisms rather than their direct clinical relevance in CD. While numerous experimental and translational studies have consistently demonstrated interactions between Mg homeostasis, inflammatory signaling, oxidative stress, mitochondrial function, and neuronal activity, these observations originate predominantly from non-CD settings [10,11,49,52,53,60,73,75,76]. Consequently, extrapolation of these mechanisms to CD should be interpreted with caution [11,64,73].

In contrast, studies performed in patients with CD have focused primarily on Mg status, dietary intake, and nutritional deficiencies, whereas direct evaluation of neuroinflammatory pathways or gut–brain axis alterations remains limited [64,69,70,71,72,73,77]. Moreover, differences in study populations, disease activity, dietary adherence, biomarker selection, and assessment methods contribute substantially to the variability observed across studies [64,70,71,77]. These methodological differences complicate direct comparisons and currently preclude definitive conclusions regarding the contribution of Mg homeostasis to neurological manifestations in CD [64,70,71,73].

The classification presented in Table 1 reflects these differences in evidentiary strength. Findings derived from mouse and rat models or from inflammatory disorders other than CD provide valuable biological insights while offering only indirect support for CD-specific pathophysiology [49,52,53,60,75,76]. Conversely, investigations conducted in CD populations offer greater clinical relevance but have largely evaluated nutritional status rather than neuroimmune mechanisms or gut–brain axis dysfunction [64,69,70,71,72,73,77]. Likewise, interventional studies remain limited, and translation of promising biological mechanisms into measurable neurological benefit has not been consistently demonstrated, as illustrated by randomized trials conducted in neurological disorders [80,81].

The current body of published research supports continued investigation of Mg as a potential modifier of inflammatory and neuroimmune pathways rather than as an independent pathogenic determinant of neurological dysfunction in CD. Future prospective studies should integrate comprehensive assessment of Mg homeostasis with intestinal barrier integrity, inflammatory biomarkers, gut–brain axis function, and standardized neurological phenotyping to determine whether alterations in Mg homeostasis contribute meaningfully to neurological manifestations in CD or primarily reflect the consequences of chronic intestinal inflammation and malabsorption [70,73,76,79].

### 5.1. Systemic Inflammation and Blood–Brain Barrier (BBB) Dysfunction

Chronic systemic inflammation is considered one of the mechanisms potentially contributing to the neurological manifestations associated with CD [6,9]. Disruption of blood–brain barrier (BBB) integrity has been proposed as one of the pathways through which intestinal inflammation may influence the CNS, although direct disease-specific evidence remains limited [9,48]. Although intestinal inflammation represents the primary site of disease activity, persistent immune activation extends beyond the gastrointestinal tract through sustained production of pro-inflammatory mediators, including IL-6, IL-1β, and TNF-α, which may promote endothelial dysfunction, increased vascular permeability, and amplification of systemic inflammatory responses [48,53].

Persistent cytokine signaling, oxidative stress, and activation of inflammatory pathways, including NF-κB and the NLRP3 inflammasome, may compromise BBB integrity by disrupting endothelial tight junctions [48,65]. Increased BBB permeability may facilitate the entry of circulating inflammatory mediators into the brain parenchyma, promoting microglial activation, neuroimmune signaling, and neuronal dysfunction [48]. Additional processes, including complement activation, coagulation imbalance, and matrix metalloproteinase activity, may further exacerbate neurovascular injury and sustain neuroinflammatory responses [48].

Within this context, experimental and translational studies suggest that Mg may contribute to neurovascular and neuronal homeostasis through several complementary mechanisms. As a physiological calcium antagonist, Mg limits excessive Ca^2+^ influx through NMDA receptors, thereby attenuating excitotoxicity, mitochondrial dysfunction, and inflammatory neuronal injury [10,82]. Mg has also been implicated in preserving BBB integrity and regulating neurotrophic signaling pathways, including brain-derived neurotrophic factor (BDNF), which plays an important role in neuronal survival, synaptic plasticity, and cognitive function [12]. Reduced Mg availability has been associated with impaired BDNF signaling and decreased neuroplasticity in experimental models. These observations support the biological rationale for a modulatory role of Mg in neuroimmune regulation; however, their relevance to neurological manifestations in CD remains to be clarified.

Conversely, reduced Mg availability has been associated with enhanced inflammatory signaling, oxidative stress, and increased production of pro-inflammatory mediators, including TNF-α, IL-1β, nitric oxide (NO), and reactive oxygen species (ROS) [12,49,56]. However, evidence regarding the relationship between Mg status and BBB dysfunction or neurological manifestations in CD derives predominantly from experimental and translational studies rather than disease-specific clinical investigations [11,14,49,82,83]. Accordingly, whether these pathways contribute directly to nervous system involvement in patients with CD remains uncertain.

Several limitations should be considered when interpreting the available evidence. Serum Mg concentrations may not accurately reflect total body Mg stores, reducing the reliability of deficiency assessment. Furthermore, published studies are heterogeneous with respect to study design, patient characteristics, disease activity, dietary adherence, biomarker selection, and the methods used to assess Mg status and dietary intake. Potential confounding factors, including generalized malabsorption, dietary quality, and coexisting conditions, may further influence Mg homeostasis and neurological outcomes in CD. Finally, the relatively small number of longitudinal and interventional studies precludes firm conclusions regarding causality and underscores the need for well-designed prospective investigations evaluating the relationship between Mg status, neuroinflammatory pathways, BBB dysfunction, and neurological manifestations in CD.

### 5.2. Microglial Activation and Neuroimmune Signaling

Microglial activation has been proposed as one of the mechanisms through which chronic systemic inflammation may contribute to neurological manifestations in CD. Persistent exposure to circulating pro-inflammatory cytokines and damage-associated molecular patterns generated during immune activation may stimulate microglia, promoting the release of TNF-α, IL-6, reactive oxygen species (ROS), nitric oxide (NO), and complement factors [84,85]. Sustained activation of these responses may contribute to synaptic dysfunction, demyelination, and neuronal injury [10].

Neuroimmune responses may be further amplified through activation of intracellular inflammasomes, particularly the NLRP3 complex, which promotes caspase-1-dependent maturation of IL-1β and IL-18 [65]. Additional innate immune receptors, including Toll-like receptors and NOD-like receptors, may further enhance cytokine production within the CNS [66]. BBB dysfunction may potentiate these processes by facilitating immune cell infiltration and the passage of circulating inflammatory mediators into the CNS [65]. Persistent immune activation has also been linked to structural brain alterations and increased circulating biomarkers of neuronal and astrocytic injury, including neurofilament light chain and glial fibrillary acidic protein (GFAP) [48].

Within this context, experimental and translational studies suggest that Mg may modulate several pathways involved in neuroimmune regulation. Adequate Mg availability contributes to intracellular Ca^2+^ homeostasis and physiological regulation of NMDA receptor activity, thereby limiting excessive excitatory signaling and downstream inflammatory responses [83,86]. Mg has also been reported to suppress NF-κB-dependent signaling and reduce the expression of pro-inflammatory cytokines, including TNF-α and IL-6 [62].

Conversely, Mg deficiency has been associated with increased intracellular Ca^2+^ influx, oxidative stress, and enhanced release of substance P (SP), a neuropeptide involved in neuroimmune communication [10,49,74,86]. Experimental findings further indicate that SP promotes microglial activation and stimulates the production of cytokines, prostaglandin E_2_, and ROS [86], while contributing to increased vascular permeability and BBB dysfunction [87,88]. Reduced Mg availability may also impair mitochondrial function, exacerbate oxidative stress, and reduce neuronal resilience. In contrast, adequate Mg status may limit ROS generation, inhibit nitric oxide synthase activity, and attenuate cytokine production [87].

Taken together, these observations suggest that Mg may act as a modulator of neuroimmune regulation through effects on calcium homeostasis, oxidative balance, and inflammatory signaling [10,53,56,62,81]. However, most available data originate from experimental models and mechanistic investigations rather than clinical studies conducted in patients with CD [49,76]. Consequently, whether disturbances in Mg homeostasis influence microglial activation or contribute meaningfully to neurological manifestations in CD remains uncertain. Well-designed prospective studies integrating comprehensive Mg assessment with neuroinflammatory biomarkers and standardized neurological evaluation are required to clarify the clinical relevance of these pathways in celiac disease.

### 5.3. Gut–Brain Axis Dysfunction and Neuroinflammatory Pathways in Celiac Disease

The gut–brain axis is a bidirectional communication network linking the gastrointestinal tract and the CNS through interconnected neural, immune, endocrine, and microbial pathways [89,90,91,92,93,94,95]. Increasing evidence indicates that alterations in gut microbiota composition and function influence neuroimmune communication through interactions involving intestinal barrier integrity, immune activation, microbial metabolites, and neural signaling [89,90,91,92]. In CD, chronic intestinal inflammation, epithelial barrier dysfunction, and gut microbiota dysbiosis may disrupt this network, thereby promoting systemic immune activation and neuroinflammatory responses [48,53,93,94,95,96].

**Figure 1:**
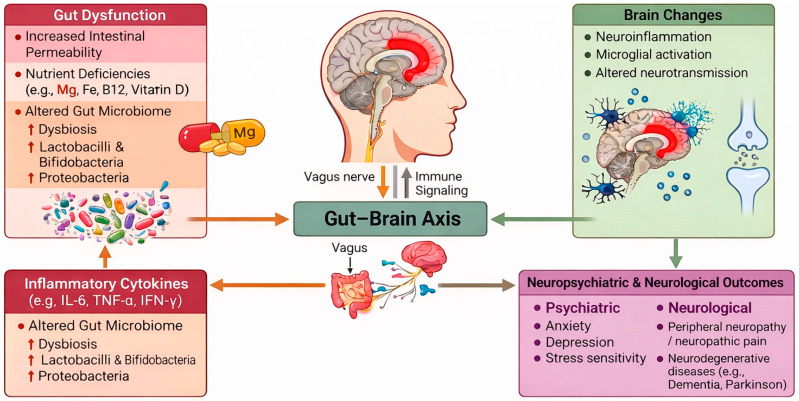
Gut–brain axis dysfunction in celiac disease. Chronic intestinal inflammation, epithelial barrier disruption, gut microbiota dysbiosis, and altered Mg homeostasis may collectively contribute to systemic immune activation and pro-inflammatory cytokine release. Through interconnected immune, neural, endocrine, and microbial pathways, these processes may influence CNS signaling, inflammatory responses, and neurological function. The proposed interactions are supported predominantly by experimental and indirect evidence and should therefore be interpreted as a conceptual framework rather than established disease-specific mechanisms. Arrows indicate the direction of the proposed interactions; their colors are used only for visual differentiation and have no additional significance Created in BioRender. Popa, E. (2026) https://BioRender.com/b8kxxzk (accessed on 1 July 2026).

Persistent production of pro-inflammatory cytokines, including IL-6, TNF-α, and IL-1β, has been implicated in BBB dysfunction, microglial activation, and inflammatory signaling within the CNS, providing a biological framework linking intestinal inflammation with neurological manifestations [48,53,96]. Increased intestinal permeability may further facilitate the translocation of inflammatory mediators and microbial products into the systemic circulation, amplifying immune activation and promoting neuroinflammatory processes [97,98,99,100]. The principal interactions among intestinal inflammation, epithelial barrier dysfunction, gut microbiota alterations, systemic immune activation, and Mg homeostasis are illustrated in Figure 1.

At the molecular level, reduced expression of peroxisome proliferator-activated receptor gamma (PPARγ), an anti-inflammatory transcription factor involved in immune regulation and host–microbiota interactions, has been reported in inflammatory intestinal disorders, including CD [97,98]. Downregulation of PPARγ is thought to contribute to persistent intestinal inflammation and dysbiosis, thereby influencing interactions among the intestinal microbiota, immune cells, and enteric neurons [26,49,98,99,100,101,102,103,104]. Similar microbiota-mediated processes have been described in neurodegenerative disorders, where microbial dysbiosis and immune activation have been linked to neuroinflammation and neurological dysfunction [89,91,92,105,106]. Nevertheless, the applicability of these observations to CD remains indirect and has yet to be confirmed in disease-specific studies.

Within this framework, disturbances in Mg homeostasis may represent an additional modifier of neuroimmune communication. Chronic intestinal inflammation and epithelial barrier disruption may impair Mg absorption, thereby reducing Mg availability and potentially linking intestinal pathology, gut microbiota alterations, and neuroimmune signaling. Preclinical evidence indicates that adequate Mg levels may attenuate NF-κB activation, reduce pro-inflammatory cytokine production, and maintain cellular homeostasis [10,13,74,75,107]. Conversely, Mg deficiency has been linked to oxidative stress, disrupted calcium homeostasis, and enhanced inflammatory responses, processes that may further impair bidirectional communication between the gut and the CNS [10,102,108]. Experimental observations also suggest that Mg deficiency may influence gut microbiota composition and host–microbiota interactions, although clinical evidence specific to CD remains limited [10,108,109].

Altered Mg homeostasis may also influence transient receptor potential melastatin 7 (TRPM7), an ion channel involved in Mg transport, inflammation, and neuronal survival [110]. Although TRPM7 has been implicated in neuroimmune regulation and inflammatory signaling, its role in gut–brain axis dysfunction in CD remains unclear [109,111]. Likewise, clinical studies directly evaluating the relationship between Mg status and neurological manifestations in patients with CD are still limited [75,91,107,108].

**Figure 2:**
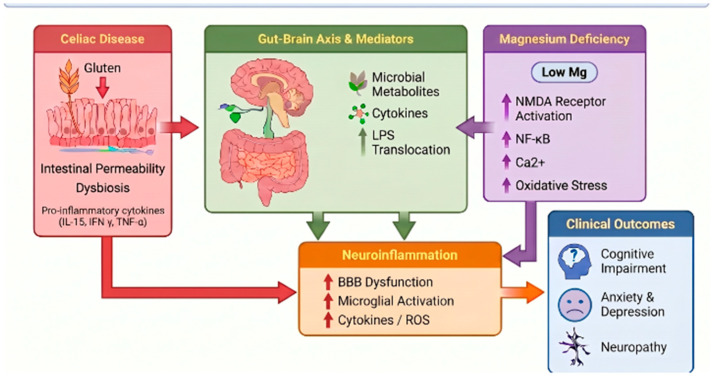
Conceptual framework illustrating the proposed interactions between Mg homeostasis, gut–brain axis dysfunction, and neuroinflammation in celiac disease. Intestinal inflammation and epithelial barrier dysfunction, together with gut microbiota alterations, may interact with Mg deficiency-associated mechanisms to influence neuroimmune signaling, neuroinflammatory pathways, and CNS homeostasis. These interactions are supported predominantly by experimental and indirect evidence and require further validation in disease-specific clinical studies. Arrows indicate the direction of the proposed interactions; their colors are used only for visual differentiation and have no additional significance. Created in BioRender. Popa, E. (2026) https://BioRender.com/b8kxxzk (accessed on 1 July 2026).

The pathways discussed above are integrated in Figure 2, which summarizes the proposed interactions among intestinal inflammation, epithelial barrier dysfunction, gut microbiota alterations, disturbances in Mg homeostasis, and neuroinflammatory signaling in CD.

## 6. Discussion

The relationship between intestinal inflammation, neuroimmune signaling, and nervous system involvement has attracted increasing attention in chronic inflammatory disorders. In CD, accumulating evidence indicates that persistent systemic inflammation and disturbances of the gut–brain axis may contribute to extraintestinal manifestations, including neurological complications [15,47,90]. Recognition of neurological involvement in CD, together with neuropathological evidence of immune-mediated nervous system injury in gluten-related disorders, supports the biological plausibility of bidirectional communication between the intestine and the nervous system [15,17]. Within this multifactorial biological framework, Mg has attracted growing interest because of its established roles in inflammatory regulation, oxidative balance, neuronal homeostasis, and cellular metabolism [10,11,53,60]. Nevertheless, the extent to which Mg contributes specifically to neuroinflammatory processes and gut–brain axis dysfunction in CD remains uncertain.

Neurological manifestations associated with CD include peripheral neuropathy, cerebellar ataxia, epilepsy, headache, cognitive impairment, and psychiatric disorders [15]. Neuropathological investigations have identified inflammatory infiltrates, immune-mediated tissue injury, and neuronal damage in gluten-related neurological disorders, supporting the concept that neurological involvement represents an important extraintestinal component of CD [17]. Current evidence indicates that intestinal inflammation, epithelial barrier dysfunction, systemic immune activation, gut microbiota alterations, and nutritional deficiencies interact within the gut–brain axis rather than acting as isolated pathogenic processes [25,90,93,109,112]. However, the relative contribution of each of these factors to neurological dysfunction has not yet been clearly defined.

Importantly, CD is a genetically determined, gluten-dependent autoimmune disease, whereas disturbances in Mg homeostasis are more likely to arise as secondary consequences of intestinal malabsorption, chronic inflammation, dietary inadequacies, or a combination of these factors [19,64,69]. Available data suggest that reduced Mg availability in CD may result from impaired intestinal absorption, persistent inflammatory activity, and suboptimal dietary intake, particularly among individuals adhering to a long-term GFD [78,113,114]. Accordingly, altered Mg homeostasis should be regarded primarily as a consequence of intestinal disease rather than an independent hallmark of CD. Whether these alterations directly influence clinically relevant neurological outcomes remains uncertain [73,94,115,116,117].

Preclinical and translational evidence supports several biologically plausible pathways through which Mg may influence neuroimmune regulation. Adequate Mg availability has been proposed to modulate excitotoxicity, intracellular calcium homeostasis, oxidative balance, mitochondrial function, endothelial integrity, and microglial activation, thereby influencing processes implicated in neuroinflammation [10,11,53,73]. Despite this strong biological rationale, translation of these findings into clinically meaningful benefits has proved challenging. Large randomized controlled trials evaluating Mg supplementation in acute stroke failed to demonstrate significant improvements in functional recovery or disability despite encouraging experimental data [80,81]. These observations highlight the limitations of directly extrapolating preclinical findings to clinical practice and emphasize that evidence supporting Mg-dependent neuroprotection in CD remains largely indirect [11,14,58,118,119].

The gut–brain axis provides a useful framework for integrating intestinal inflammation, epithelial barrier dysfunction, systemic immune activation, gut microbiota alterations, and neuroimmune signaling in CD [25,90,93,109,112]. Within this multifactorial framework, Mg is unlikely to represent the principal driver of neurological dysfunction but rather one of several interacting modulators capable of influencing inflammatory and neuroimmune responses. Consequently, the existing evidence does not permit definitive conclusions regarding the specific contribution of Mg to gut–brain axis dysfunction or neurological manifestations in patients with CD.

From a clinical perspective, Mg should therefore not be regarded as a disease-specific treatment for neurological complications of CD. Instead, optimization of Mg status should be considered part of a comprehensive management strategy centered on strict adherence to a GFD, restoration of intestinal mucosal integrity, and correction of documented nutritional deficiencies. Although maintaining adequate Mg intake contributes to overall nutritional health, evidence supporting direct neurological benefits remains limited [11,73]. In contrast, improvements in cognitive symptoms observed in some patients following initiation of a GFD appear to correlate with histological recovery and serological markers of disease control, emphasizing the importance of treating the underlying intestinal disease process [120].

An additional aspect of clinical relevance is the increasing public interest in the relationship between intestinal health, gut–brain axis dysfunction, gluten exposure, and Mg supplementation. In routine clinical practice, patients presenting with nonspecific neurological symptoms, including fatigue, headache, cognitive complaints, anxiety, or mood disturbances, may adopt a gluten-free diet without prior evaluation for CD or other gluten-related disorders. Likewise, Mg supplements are frequently used empirically in an attempt to alleviate similar symptoms despite the absence of documented Mg deficiency or established clinical indications. At present, available evidence does not support recommending either a GFD or Mg supplementation for nonspecific neurological symptoms in the absence of an appropriate diagnostic evaluation. Furthermore, unnecessary adoption of a GFD may itself reduce dietary Mg intake because many commercially available gluten-free products contain lower amounts of Mg than their gluten-containing counterparts [72,73]. These considerations highlight the importance of accurate diagnosis, individualized nutritional assessment, and evidence-based clinical decision-making before recommending dietary restrictions or nutritional supplementation.

Taken together, the current body of evidence does not support a causal relationship between altered Mg homeostasis and neurological manifestations in CD. Reduced Mg availability is more likely to reflect the consequences of intestinal dysfunction, chronic inflammation, and dietary factors than to represent an independent pathogenic mechanism. Although Mg may modulate inflammatory and neuroimmune pathways relevant to gut–brain axis function, the clinical significance of these effects remains to be clarified through disease-specific investigations.

Future prospective studies, including longitudinal and interventional investigations, should determine whether correction of Mg deficiency influences neurological outcomes, inflammatory biomarkers, intestinal barrier integrity, or gut–brain axis-related mechanisms in patients with CD [70]. Integration of nutrigenomics, metabolomics, microbiome profiling, comprehensive Mg assessment, and detailed neurological phenotyping may improve understanding of interindividual variability in Mg metabolism and inflammatory responses, although the clinical applicability of these approaches requires further validation [121,122]. Studies integrating assessment of gut microbiota composition, intestinal permeability, systemic inflammation, Mg homeostasis, and neurological function may further clarify the contribution of Mg to the multifactorial pathophysiology of CD [73,76,90,108,109,122,123]. Such integrated approaches may also help define the potential role of Mg in precision medicine strategies for patients with CD.

## 7. Conclusions

This review integrates the current understanding of Mg homeostasis, neuroinflammatory pathways, and gut–brain axis dysfunction in celiac disease. Mg appears to act as a modulator of inflammatory and neuroimmune processes rather than an independent pathogenic mechanism.

Current knowledge supports a multifactorial model in which chronic intestinal inflammation, epithelial barrier dysfunction, gut microbiota alterations, systemic immune activation, and nutritional factors collectively contribute to neurological involvement in CD.

Within this complex pathophysiological context, Mg status is more likely to reflect the consequences of intestinal malabsorption, persistent inflammation, and suboptimal dietary intake while potentially influencing neuroimmune regulation. Experimental and translational studies provide biological support for these mechanisms, but confirmation in disease-specific clinical investigations is still required.

Routine Mg supplementation cannot currently be recommended for neurological symptoms or presumed gut–brain axis dysfunction in patients with CD in the absence of documented Mg deficiency or other established clinical indications. Maintenance of adequate Mg status should instead be considered within a comprehensive nutritional strategy that includes strict adherence to a GFD and correction of confirmed micronutrient deficiencies.

Future research should combine comprehensive assessment of Mg status with evaluation of intestinal barrier integrity, gut microbiota composition, inflammatory biomarkers, and standardized neurological phenotyping. This multidisciplinary approach may improve understanding of the role of Mg within the multifactorial interplay between intestinal inflammation, neuroimmune regulation, and gut–brain axis dysfunction in CD.

## Figures and Tables

**Table 1:** Levels of evidence and translational relevance of Mg-related mechanisms in celiac disease.

Evidence Type	Domain	Main Findings	Strength of Evidence	Relevance to Celiac Disease
Experimental (mouse and rat models)	Mg and neuroinflammation	Mouse and rat models demonstrate that Mg deficiency, or conversely Mg supplementation, modulates oxidative stress, neurogenic inflammation, NMDA receptor–mediated glutamatergic signaling, glial activation, neuronal oscillations, synaptic plasticity, cognitive function, and pain hypersensitivity, supporting a mechanistic role for Mg in neuroinflammatory pathways [49,52,53,60,75,76]	Moderate (mechanistic evidence)	Indirect (non-CD models)
Translational studies	Mg and neurovascular/neuroimmune mechanisms	Experimental and translational evidence suggests that Mg may influence BBB-related responses and neuroimmune signaling, with potential implications for neuroinflammatory pathways [12]	Low	Indirect
Observational studies (cross-sectional, CD populations)	Mg status in celiac disease	Heterogeneous findings regarding Mg deficiency prevalence, influenced by biomarker limitations and study design variability [71,77]	Low–moderate	Direct
Nutritional studies	Mg intake in gluten-free diet	Reduced magnesium intake and broader micronutrient inadequacies have been reported in individuals following GFD, potentially related to dietary composition and low mineral content [69,78,79]	Moderate	Direct
Clinical studies (interventional/longitudinal)	Mg and neurological outcomes	Clinical evidence remains limited, with large, randomized trials in neurological conditions showing neutral or inconclusive results despite strong mechanistic rationale [80,81]	Very low	Direct/indirect
Review and integrative literature	Mg and brain function	Literature describes potential mechanistic links between Mg homeostasis and neuroinflammatory pathways; however, the available evidence is largely indirect and not specific to CD [10,11,64,73,78,79]	Low	Indirect

## Data Availability

No new data were created or analyzed in this study. Data sharing is not applicable to this article.

## Abbreviations

The following abbreviations are used in this manuscript:

ACC	Anterior Cingulate Cortex
ACE-R	Addenbrooke’s Cognitive Examination-Revised
ADHD	Attention deficit hyperactivity disorder
ASD	Autism spectrum disorder
ATP	Adenosine triphosphate
ATPase	Adenosine triphosphatase
BBB	Blood–brain barrier
BDNF	Brain-derived neurotrophic factor
CD	Celiac disease
CNS	Central nervous system
CRP	C-reactive protein
GAD	Glutamic acid decarboxylase
GFAP	Glial fibrillary acidic protein
GFD	Gluten-free diet
IFN-γ	Interferon gamma
IL	Interleukin
Mg	Magnesium
MLCK	Myosin light chain kinase
MMSE	Mini-Mental State Examination
MoCA	Montreal Cognitive Assessment
NCGS	Non-celiac gluten sensitivity
NF-κB	Nuclear factor kappa B
NLRP3	NOD-like receptor family pyrin domain containing 3
NO	nitric oxide
NMDA	N-methyl-D-aspartate
PAR-2	Protease-activated receptor 2
PPARγ	Peroxisome proliferator-activated receptor gamma
RAVLT	Rey Auditory Verbal Learning Test
ROCF	Rey–Osterrieth Complex Figure
ROS	Reactive oxygen species
SOD	Superoxide dismutase
TG2	Tissue transglutaminase 2
TG6	Tissue transglutaminase 6
TFH	T follicular helper
TJ	Tight junction
TNF-α	Tumor necrosis factor alpha
TRPM6	Transient receptor potential melastatin 6
TRPM7	Transient receptor potential melastatin 7
ZO-1	Zonula occludens-1
